# Benchmarking MS/MS Featurization Strategies for Machine Learning-Driven Metabolite Structure Annotation

**DOI:** 10.1021/jasms.5c00428

**Published:** 2026-06-15

**Authors:** Roger Giné, Ivan Pérez-López, Josep M Badia, Jordi Capellades, Oscar Yanes

**Affiliations:** † 16777 Universitat Rovira i Virgili, Department of Electronic Engineering, 43007 Tarragona, Spain; ‡ CIBER de Diabetes y Enfermedades Metabólicas Asociadas (CIBERDEM), Instituto de Salud Carlos III, 28029 Madrid, Spain; § Metabolomics Platform, Institut de Recerca Biomèdica Catalunya Sud, Hospital Universitari Sant Joan de Reus, 43204 Reus, Spain

**Keywords:** untargeted metabolomics, tandem mass spectrometry, metabolite annotation, spectral featurization, machine learning, structure retrieval

## Abstract

Reference MS/MS libraries remain incomplete due to the vast chemical diversity of metabolites, leaving many spectra from untargeted metabolomics experiments unannotated—the “dark matter” of metabolomics. Machine learning can extend metabolite annotation beyond direct library matches, but its success depends critically on how MS/MS spectra are converted into numerical representations that capture chemically meaningful features while reducing sparsity. Although numerous spectral representations exist, they have not been systematically compared. Using over 71,000 unique compounds with merged-energy MS/MS spectra, we benchmarked a broad set of spectral featurization methods, including fixed and adaptive binning, global-quantile variable-width bins, frequent-peaks representations, spectrum hashing, and learned embeddings such as Spec2Vec, MS2DeepScore, DreaMS, and SpecEmbedding. We further evaluated how vector dimensionality affects performance. A total of 105 neural network models were trained under 5-fold cross-validation to predict Mol2Vec molecular embeddings and retrieve correct structures from a 0.6-million-compound database. Retrieval was assessed at 0.1, 3, and 10 ppm mass tolerances, and a null ranking model was generated to determine expected Top-N accuracy under random candidate ordering. Adaptive binning, frequent-peaks, and DreaMS produced the most accurate embedding predictions. On the test data set, Top-1 retrieval reached 46%, 44%, and 38% for 0.1, 3, and 10 ppm, respectively, with Top-5 accuracies up to 77%. In the CASMI2022 data set, Top-1 performance remained similar at 0.1 ppm but dropped markedly at wider tolerances, reaching only 26% at 3 ppm and 23% at 10 ppm. To ensure reproducibility and broad community applicability, results were further validated on two fully open benchmark data sets, MassSpecGym and Spectraverse, with findings consistent across all three resources. These results underscore clear performance differences among featurization strategies, the strong dependence of retrieval accuracy on mass precision, and the need for evaluation metrics aligned with structure-level annotation tasks.

## Graphical abstract

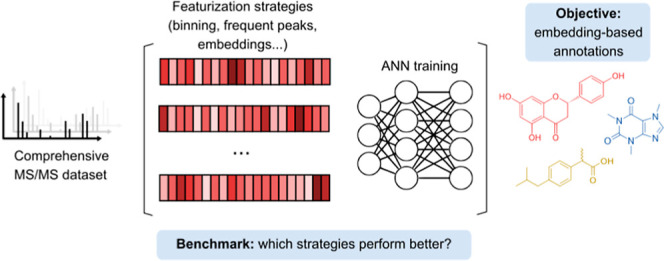

## Introduction

The interpretation of tandem mass spectrometry (MS/MS) data is central to metabolite annotation, a key step in metabolomics workflows that underpin a wide range of applications in biomedicine, nutrition, environmental science, and chemical ecology.^1^ Despite major advances in mass spectrometric instrumentation and data acquisition strategies, the confident identification of metabolites remains a significant bottleneck in untargeted metabolomics.

The predominant strategy for metabolite annotation relies on spectral library matching, where experimental MS/MS spectra are compared against reference spectra of known compounds.^2–4^ While this approach is well established and implemented in widely used resources such as NIST,^5,6^ GNPS,^7^ METLIN,^8^ MassBankEU,^9,10^ MoNA,^9^ or MS^*n*^Lib,^11^ it suffers from fundamental limitations. Reference libraries remain far from comprehensive due to the immense chemical space of metabolites, the limited commercial availability of standards, and the heterogeneous acquisition conditions used across laboratories and instruments.^12^ As a result, a large fraction—often the majority—of MS/MS spectra acquired in untargeted metabolomics experiments remain unannotated, contributing to what is often referred to as the “dark matter of metabolomics” and the “dark metabolome”.^13^

Machine learning (ML) has emerged as a promising alternative to overcome these limitations by enabling metabolite annotation beyond direct library matches.^14^ ML-based approaches to metabolite annotation span a broad methodological landscape, including fragmentation-based tools that explicitly model fragmentation chemistry to predict molecular fingerprints or formulas (e.g., SIRIUS^15^); spectral similarity–based methods that leverage learned representations to extend library matching to structurally related but unlibrarised compounds (e.g., MS2Query,^16^ MS2DeepScore^17^); spectra-to-structure annotation via learned representations, in which MS/MS spectra are encoded into numerical feature vectors or embeddings and ranked against a database of candidate molecular structures represented in the same space (e.g., ChemEmbed^18^); and de novo structure generation, in which molecular structures are generated directly from spectral data without reference to a pre-existing candidate database (e.g., MSnovelist^19^). Nevertheless, one of the central challenges in applying machine learning to MS/MS data is the inherently high-dimensional, sparse, and irregular nature of mass spectral features. Raw MS/MS spectra, commonly represented as lists of *m*/*z*–intensity pairs, vary in length and density, and exhibit large dynamic ranges in intensity, making them unsuitable for direct use as input to most machine learning models.

To enable efficient computation and robust learning, MS/MS spectra must therefore be transformed into numerical formats that preserve chemically meaningful information while reducing sparsity and dimensionality. A wide range of feature extraction strategies and spectral representations methods has been proposed, including simple binning schemes,^18^ feature hashing,^20^ spectral embeddings,^21^ peak selection heuristics,^22^ fragmentation trees,^15,23,24^ or learned representations such as spectrum transformers.^25,26^ However, despite their centrality to ML-based metabolite annotation, these preprocessing strategies have rarely been systematically compared.

In this work, we present a systematic analysis of the extent to which different MS/MS featurization strategies preserve structural information relevant for machine learning–based metabolite annotation. Building on our recently developed ChemEmbed framework, which predicts Mol2Vec^27^ molecular embeddings directly from MS/MS spectra^18^ for spectra-to-structure retrieval via shared embedding spaces, we use this task as a controlled setting to evaluate how effectively various spectral featurization strategies encode molecular features. Our central hypothesis is that representations enabling more accurate Mol2Vec prediction are those that best align with the underlying molecular similarity structure, thus being easier for machine learning models to learn from. To test this, we benchmark spectral encoding strategies across three different test data sets, including MassSpecGym and Spectraverse, and the CASMI2022 challenge, using multiple performance metrics. This approach provides practical guidance for selecting feature extraction methods, highlights how the choice of MS/MS featurization directly influences model performance, and identifies approaches that could serve as robust starting points for future ML-driven metabolomics tools.

## Experimental Section

### Tandem MS Database Curation

Positive-ion mode MS/MS spectra were obtained from four major repositories: NIST2023, Agilent-METLIN, GNPS2, and MSDIAL. Only spectra annotated as [M + H]^+^ were retained. To unify entries originating from different databases, compounds were grouped using the first 14 characters of the InChIKey, ensuring that isomeric variants shared a unique compound identifier. When multiple sources contributed spectra for the same compound, we prioritized entries according to data quality and curation depth in the following order: NIST2023 → Agilent-METLIN → GNPS2 → MSDIAL. To avoid data leakage into the CASMI benchmarking experiment, all spectra whose InChIKey (14-character prefix) matched any CASMI2022 target compound were removed.

### Spectrum Preprocessing and Merging

For each compound ID, MS/MS spectra acquired at different collision energies were merged into a single composite spectrum. Before merging, all spectra were (i) binned at 0.01 Da resolution, (ii) aligned by *m*/*z*, and (iii) combined by computing the mean intensity across spectra. Peak intensities were scaled to a 0–100 range dividing by the maximum observed intensity and multiplying by 100. The curated data set was exported in MGF format for compatibility with downstream spectral featurization strategies.

### CASMI2022 Data Set Curation

CASMI2022 raw data (mzML files and metadata) were downloaded from the official CASMI Web site (https://fiehnlab.ucdavis.edu/casmi). Only positive-mode spectra were considered. Files were processed in R (v4.5.0) using the Spectra package (v1.18.0). MS/MS scans were retrieved by matching each compound’s reference retention time (±10 s) and precursor *m*/*z* (±20 ppm). When multiple spectra were detected for a compound, the five scans with the highest precursor intensity were selected and merged into a consolidated spectrum. Final intensities were normalized to a maximum of 100, and spectra were exported to MGF format.

### MassSpecGym and Spectraverse Data Set Curation

The MassSpecGym^28^ data set was downloaded from the Polaris Hub (https://polarishub.io/datasets/roman-bushuiev/massspecgym) and the Spectraverse data set (version 1.0.1) from Zenodo (10.5281/zenodo.17870921). For Spectraverse,^29^ the MGF file was parsed using matchms v0.32.0 and compound metadata was merged with the spectral data into a pandas DataFrame. Both data sets were subsequently processed identically: spectra were subset to positive ionization mode [M + H]^+^ adduct entries, *m*/*z* values were discretized at 0.01 Da resolution, and intensities were scaled to a 0–100 range by dividing by the maximum observed intensity per spectrum and multiplying by 100. To avoid data leakage into the CASMI benchmarking experiment, all spectra whose InChIKey (14-character prefix) matched any CASMI2022 target compound were removed.

### Spectral Featurization Strategies

#### Uniform Binning

Following the ChemEmbed framework,^18^ spectra were restricted to the 0–700 *m*/*z* range and discretized using 0.01 Da fixed-size bins (i.e., feature resolution), yielding a 70,000-dimensional vector. Intensities were binarized (1 if intensity >0; otherwise 0).

#### Adaptive Binning

Used quantiles of the global *m*/*z* distribution of the entire tandem MS database to define variable-width bins. Two configurations were evaluated: (i) 1000 quantiles → 927 effective bins, and (ii) 5000 quantiles → 3621 effective bins. Quantiles with identical *m*/*z* values, corresponding to common peaks, were merged. Bin boundaries were saved for application to external data sets.

#### Frequent-Peaks Representation

Across ∼7.5 million *m*/*z* measurements, 112,232 unique peaks were detected. Peaks were ranked by frequency, and the top 500, 1000, 3000, and 5000 were selected. For each spectrum, peaks matching this list were retained, producing vectors of dimension N. Two versions were generated: (i) Top N: intensities preserved, (ii) Top N Binarized: intensities converted to 0/1 values.

#### ANN-SoLo Spectral Hashing

Spectral hashing was performed using ANN-SoLo^20^ v0.3.3 with hashed vector lengths of 100, 300, 500, 1000, 3000, and 5000. Parameters were: *m*/*z* range between 30–2000 Da, bin size = 0.01 Da, min_intensity = 0.0001. Default configuration settings were used for all other parameters.

#### Spec2Vec Embeddings

Spec2Vec^21^ (v0.9.1) embeddings were computed using the pretrained model 150225_Spec2Vec_pos_CleanedLibraries. Spectra were preprocessed according to recommended settings: *m*/*z* range = 30–3000 Da, min_intensity = 0.01, neutral loss range = 10–200 Da, intensity weighting power = 0.5, allowed missing percentage = 20%. The resulting vectors were 300-dimensional.

#### DreaMS Embeddings

DreaMS^25^ v1.0.0 embeddings were generated using the default pretrained model and the dreams_embeddings API. The resulting vectors were 1024-dimensional.

#### SpecEmbedding Embeddings

SpecEmbedding^26^ (commit c16205c) was run with a maximum tokenizer length of 100 and the SiameseModel architecture. Embeddings were inferred in batches of 512 and were 512-dimensional.

#### MS2DeepScore Embeddings

MS2DeepScore^17^ (version 2.7.2) embeddings were generated using the latest model trained with both positive and negative ionization spectra available at 10.5281/zenodo.17826815. The resulting vectors were 500-dimensional.

Note that the dimensionality was varied only for featurization strategies where this could be achieved without retraining; specifically, those for which output dimensionality is a configurable parameter of the featurisation procedure (adaptive binning, Top N, Top N binarized, and ANN-SoLo). For learned embeddings (Spec2Vec, MS2 DeepScore, Spec Embedding, and DreaMS), results reflect the dimensionalities of their respective pretrained releases.

### Artificial Neural Network (ANN) Model Training

The models were implemented in PyTorch v2.3.0a0. Each spectral representation generated an input vector of size *N*, which was fed into a fully connected feed-forward network with the architecture: *N* (input-dependent) → 2048 → 1024 → 300. All hidden layers used ReLU activation and 25% dropout to reduce overfitting. The final 300-dimensional layer represented the target Mol2Vec embedding for each compound. Model optimization was performed using a mean-square error (MSE) loss between the predicted and reference Mol2Vec vectors. We used the Adam optimizer with a learning rate of 2 × 10^–4^ throughout training. An early stopping criterion halted training when no meaningful improvement (>0.01 reduction in average test-set MSE) was observed for 10 consecutive epochs.

A 5-fold cross-validation scheme was applied using Scikit-learn (v1.5.2) with a fixed random seed and shuffled spectra to minimize biases in the gradient calculation. Across all featurization strategies and dimensionalities, this resulted in 105 trained ANN models.

To confirm the absence of chemical class bias across data partitions, compound classifications were retrieved by matching InChIKey identifiers (first 14 characters) against a publicly available InChIKey-deduplicated ClassyFire/ChemOnt^30^ annotation collection comprising more than 73 million unique compounds (https://zenodo.org/records/20108565). Annotations were successfully retrieved for 71,553 of the 71,561 compounds in the data set.

### Reference Mol2Vec Database

Mol2Vec embeddings (300-dimensional) were computed for all molecules in both the training/test databases and the CASMI2022 data set. These entries were appended to the 0.52 M structure database used previously by ChemEmbed.^18^ The final reference library contained 606,097 structures; each assigned a unique embedding.

### Candidate Retrieval and Database Matching

For each predicted molecular embedding, database candidates were filtered by exact mass at three tolerances: 0.1, 3, and 10 ppm. Cosine similarities between the predicted embedding and all candidate Mol2Vec embeddings were then computed, and candidates were ranked by decreasing similarity. Retrieval accuracy was determined by checking whether the correct structure (same 14-character InChIKey) appeared in the Top-1, Top-3, Top-5, or Top-10 positions.

### Null Distribution Estimation

To contextualize retrieval performance, a null (random) ranking model was generated for each data set. For every query spectrum, candidate molecules from the reference Mol2Vec database were randomly permuted, and the rank of the correct candidate was recorded. This was repeated 10,000 times, yielding expected retrieval probabilities for Top-1, Top-3, Top-5, and Top-10 performance under random chance.

## Results

### Construction of a MS/MS Spectral Database and Spectral Features

To create a robust foundation for evaluating spectral featurization strategies, we compiled a comprehensive MS/MS spectral database by integrating spectra from multiple sources, including NIST23, Agilent-METLIN, GNPS2, and MSDIAL. For each compound, spectra acquired at multiple collision energies were merged into a single consolidated spectrum, maximizing the amount of fragment information available for downstream molecular embedding prediction. This approach was adopted based on findings from our recent publication, ChemEmbed,^18^ in which merged spectra were shown to provide a richer and more information-dense input representation than individual collision energy spectra, yielding predicted embeddings that more closely aligned with ground-truth Mol2Vec embeddings and improved compound ranking performance. The resulting database comprises 71,561 unique compounds, each represented by a merged-energy spectrum. We only considered positive ionization mode and [M + H] adduct. Since many compounds were annotated across different reference libraries, we calculated the overlap by matching the first 14 characters of their InChIKey identifiers. To resolve duplicates, we prioritized the libraries in the following order (higher to lower priority): NIST2023 → Agilent-METLIN → GNPS2 → MSDIAL.

We then processed these spectra using a diverse set of feature extraction methods to generate machine-learning-ready vectors (Figure 1). These include: (1) Binning, where *m*/*z* values are mapped to fixed-size bins; (2) Adaptive binning, which uses variable-width bins defined by a global *m*/*z* quantile distribution; (3) Top N/Top N binarized, which select the most frequently occurring *m*/*z* bins across spectra; (4) ANN-SoLo: a spectrum hashing–based representation;^20^ and (5) Spectral embedding methods, including Spec2Vec^21^ and MS2DeepScore,^17^ and the transformer-based encoders DreaMS^25^ and SpecEmbedding.^26^

**Figure 1:**
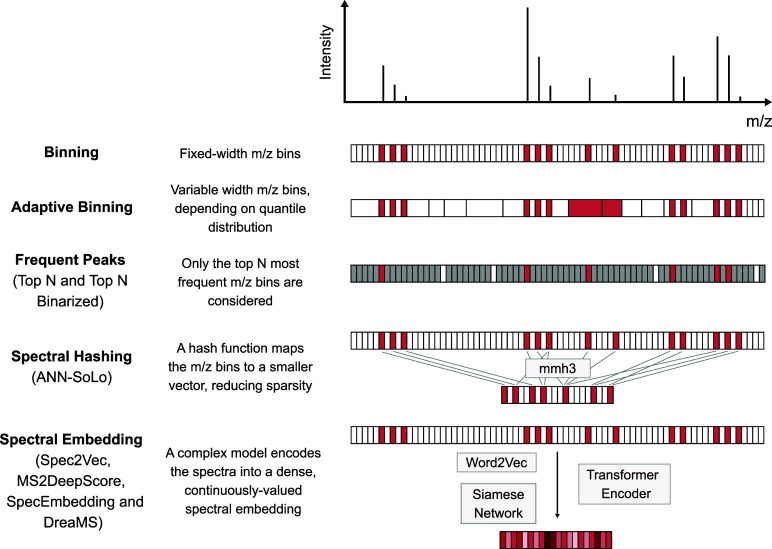
Overview of the feature extraction methods from raw MS/MS spectra compared in this study. From top to bottom: Conventional Binning divides the continuous *m*/*z* axis into fixed-size bins of 0.01 Da and assigns each peak to its corresponding bin. Adaptive Binning uses variable-width bins defined by the global *m*/*z* quantile distribution of a large spectral data set (here, our train/test data set), yielding coarse bins in sparse *m*/*z* regions and finer bins where signals are dense. In the Frequent Peaks approach, the same global *m*/*z* distribution is used to identify the top *N* most frequent *m*/*z* signals, which form a reference list for subsetting spectra. Spectral Hashing applies a cryptographic hash function (e.g., MurmurHash3, mmh3) to map conventional bins into a fixed-length hashed vector, producing a denser representation. Finally, Spectral Embedding methods use machine learning models—such as Word2Vec-based Spec2Vec, Siamese neural networks like MS2DeepScore or transformer encoders like DreaMS and SpecEmbedding—to convert spectra into dense, continuous embeddings.

In addition to comparing featurization strategies, we evaluated the effect of input vector dimensionality on model performance. For methods where dimensionality could be adjusted, we tested: varying the number of quantiles (Adaptive binning), the number of top-frequent peaks (Top N and Top N binarized), and the size of hashed vectors (ANN-SoLo). In total, the experiments encompassed 21 unique method–dimension combinations, enabling a comprehensive assessment of how feature extraction strategies and vector size influence the ability to capture molecular information.

### Evaluation of MS/MS Spectral Representations on ANN-Based Molecular Embedding Prediction

To assess how well each spectral representation method supports Mol2Vec embeddings prediction, we trained artificial neural networks (ANNs) under a unified cross-validation framework. The MS/MS database was randomly partitioned into five equally sized splits (*N* = 14,312 spectra each). Using 5-fold cross-validation, models were trained on four splits and evaluated on the remaining one, resulting in a total of 105 ANN models across all spectral representation methods and vector dimensionalities. To confirm the absence of systematic distributional bias across partitions, we examined the precursor mass and chemical (super)­class distribution for each fold. The distributions were highly consistent across all five folds, with no evidence of systematic enrichment or depletion in any individual partition (Figure S1).

To balance model expressivity, training speed, and overfitting control, we implemented a compact ANN architecture (Figure 2a). Only the input layer varied in size to match each representation’s dimensionality, while all subsequent layers were kept constant across models. Training was performed with early stopping, and all models converged between 40 and 85 epochs (Figure 2b). For each fold, the best model was defined as the one achieving the lowest mean-squared error (MSE) on the held-out split (Figure 2c).

**Figure 2:**
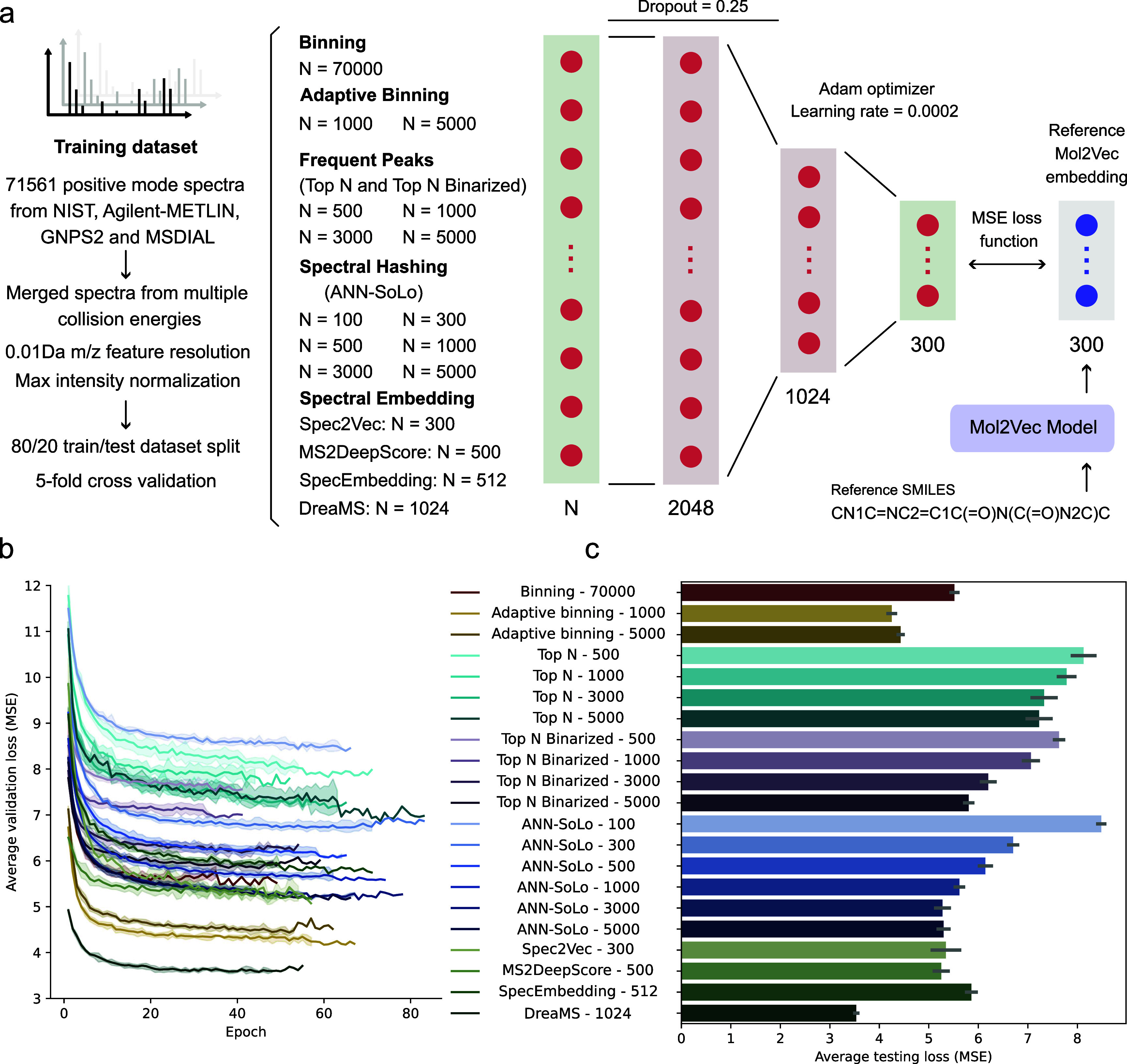
(a) Neural network architecture and input representations: The data set consisted of 71,561 positive-ion MS/MS spectra from NIST23, Agilent-METLIN, GNPS2, and MSDIAL, merged across collision energies. Spectra were preprocessed (0.01 Da feature resolution and intensity normalization) and split into five folds for cross-validation. Spectral representation methods and corresponding vector sizes included: conventional Binning (*N* = 70,000); Adaptive Binning (*N* = 1000 and 5000); Top N most frequent peaks with binarized or nonbinarized intensities (*N* = 500, 1000, 3000, 5000); ANN-SoLo spectrum hashing (*N* = 100, 300, 500, 1000, 3000, 5000); and the spectral embedding models Spec2Vec (*N* = 300), MS2DeepScore (*N* = 500), SpecEmbedding (*N* = 512) and DreaMS (*N* = 1024). For each representation, an ANN was trained using a common architecture: an input layer of size N with 0.25 dropout, followed by layers of 2048 units (0.25 dropout), 1024 units, and a 300-unit output layer. Models were optimized using mean-squared error (MSE) to regress reference Mol2Vec embeddings (300-dimensional) computed from SMILES for all 71,561 compounds. (b) Model performance: All models converged between 40 and 85 epochs. The plot shows the average test-set MSE across the five cross-validation folds, with shaded regions representing the standard deviation. (c) For each fold, the best model was defined as the one achieving the lowest test-set MSE on the held-out split. Error bars represent the MSE of these best-performing models for each spectral representation method.

Overall, the best-performing models—those achieving the lowest test MSE across the cross-validation runs—were trained using tandem mass spectra encoded with DreaMS and Adaptive Binning (both 1000 and 5000 dimensions). A general trend was observed across methods: increasing vector dimensionality improved model performance up to a saturation point (e.g., ANN-SoLo at 3000 dimensions), after which additional dimensions offered no clear benefit. For the Frequent Peaks method, binarizing peak intensities consistently improved performance relative to nonbinarized versions, particularly at higher dimensions (Top 3000 and 5000). Notably, Spec2Vec, despite having an input size of only 300 dimensions, achieved MSE values comparable to much larger representations such as 70,000-dimensional Binning and 3000-dimensional ANN-SoLo, illustrating the effectiveness of dense embedding approaches.

### Benchmarking Model Performance on the Held-Out Test Data Set

We next evaluated how well each model predicted molecular embeddings on the unseen test data set using three complementary criteria: (i) alignment between predicted and true Mol2Vec embeddings (cosine similarity), (ii) spatial proximity in the embedding space (Euclidean distance), and (iii) retrieval accuracy of the correct molecular structure from a large chemical reference database.

Cosine similarities between predicted and true Mol2Vec vectors were generally high and more uniform across models than the MSE values, though the same global performance trends remained evident (Figure 3a). Median cosine similarity scores for all methods were close to or above 0.95—corresponding to only an 18° angular deviation between predicted and true embeddings. By contrast, Euclidean distances (Figure 3b) displayed greater variability across models, closely mirroring their relative MSE performance.

**Figure 3:**
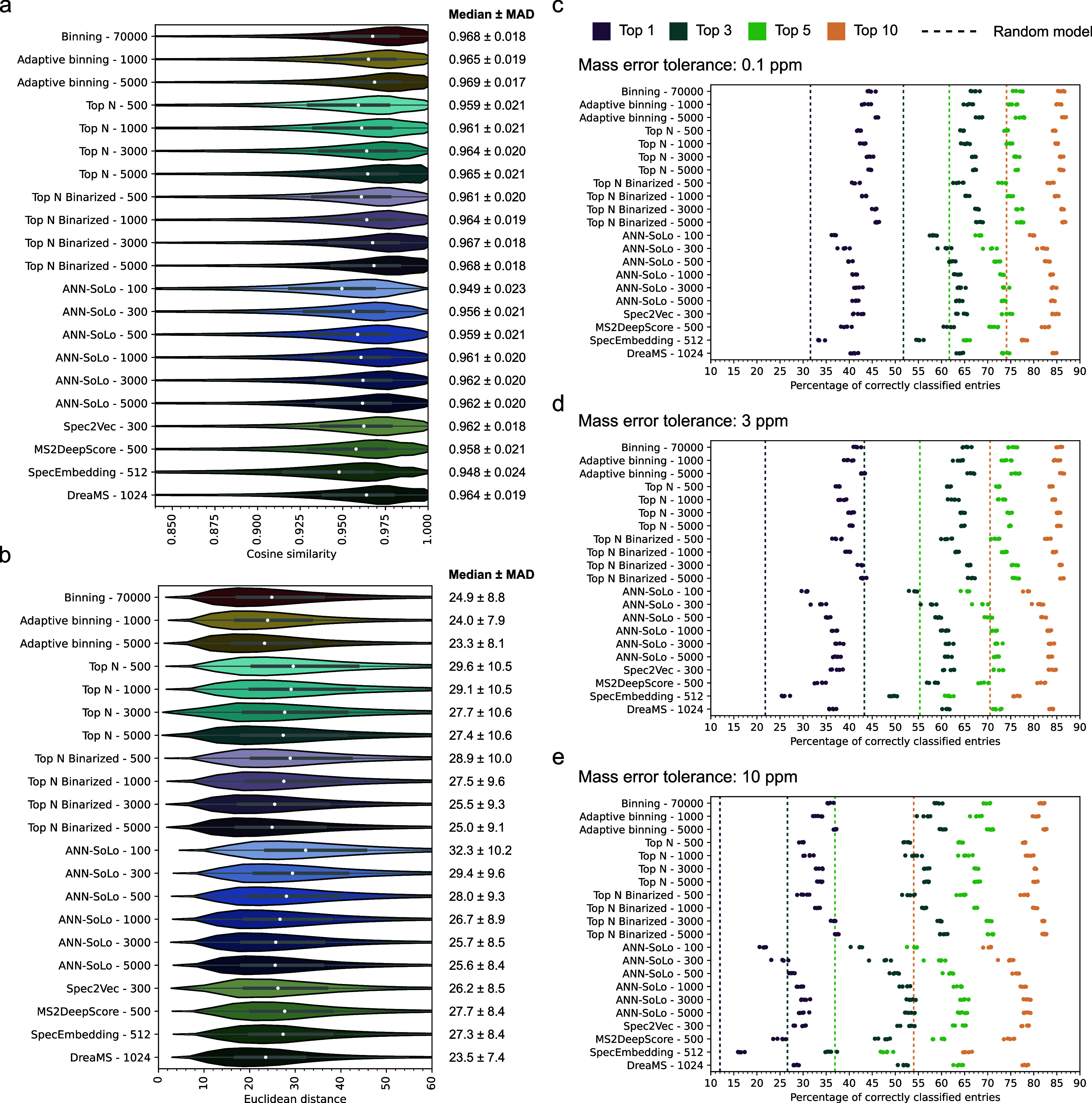
Performance evaluation on the held-out test data set. For each spectral representation, all five cross-validation models were evaluated using their corresponding test split (*N* = 14,312), which was not used during training. (a,b) Distributions of cosine similarity (a) and Euclidean distance (b) between the predicted and true Mol2Vec embeddings; median and median absolute deviation (MAD) included. (c–e) Retrieval performance when identifying the correct molecular structure from a reference database of ∼0.6 million unique molecules. After filtering candidates by exact mass within (c) 0.1 ppm, (d) 3 ppm, or (e) 10 ppm, we ranked all candidates by cosine similarity to the predicted Mol2Vec embedding and recorded the frequency with which the correct structure (same 14-character InChIKey) appeared in the Top-1, Top-3, Top-5, or Top-10 positions. As a baseline, dashed lines indicate the expected retrieval percentages for a random ranking model, accounting for the varying number of candidates at each ppm tolerance.

To assess the functional usefulness of the predicted embeddings, we evaluated each model’s ability to retrieve the correct molecular structure—defined by an identical 14-character InChIKey—from a large reference set containing over 0.60 million molecular structures. This database included the full training/test data set as well as all candidates from the CASMI 2022 challenge.

We simulated varying mass accuracy conditions by constraining candidate lists to three mass error tolerances: 0.1, 3, and 10 ppm, reflecting the expected mass precision of FT-ICR, Orbitrap, and qTOF instruments, respectively (Figure 3c–e). Varying the ppm tolerance directly modulates the number of candidate structures retrieved from the 0.60 million structures database (Figure S2) and therefore reduced the probability of retrieving the correct structure. To establish a baseline, we computed a random ranking model that estimates the expected Top-N accuracy when candidate ordering is random—thus accounting for the fact that narrower ppm windows inherently increase the chance of success due to fewer candidates.

Top-1 retrieval rates peaked at 46%, 44%, and 38% for 0.1, 3, and 10 ppm, respectively, while Top-5 performance reached 77%, 76%, and 71%. Notably, although accuracy decreased slightly with more permissive mass windows, the difference relative to the random model increased. This indicates that as chemically distinct candidates are added to the search space (nonisomeric structures), the predicted embeddings remain sufficiently specific to identify the correct molecule.

Overall, retrieval performance generally mirrored the MSE trends, with two important exceptions. First, DreaMS, despite having one of the lowest average MSE values, performed similarly to midperforming models such as Spec2Vec, MS2DeepScore and ANN-SoLo (3000 and 5000), indicating that its low MSE does not fully translate into better structure-level discrimination. Second, SpecEmbedding showed the opposite pattern: although its MSE was comparable to top-performing methods (e.g., Top-N Binarized 3000), it produced markedly lower cosine similarities, higher Euclidean distances, and retrieval rates only ∼5% above the random baseline. These discrepancies indicate that MSE alone does not fully capture the qualitative properties of predicted Mol2Vec embeddings that are most critical for molecular retrieval, highlighting the necessity of complementary performance metrics.

### Benchmarking Model Performance on the CASMI2022 Data Set

Next, we applied the same benchmarking analysis to the CASMI2022 challenge data set, which includes 304 molecular structures absent from the training and test sets. As expected, performance declined across all models relative to the held-out test data. This reduction is likely due to the fact that CASMI spectra were acquired at a single collision energy, whereas our models were trained on merged multienergy spectra, which contain substantially richer fragmentation information. With less detail available, cosine similarity values decreased to 0.92–0.95 (Figure 4a), corresponding to vector angles of roughly 23–18 degrees. The drop in performance was even more pronounced for Euclidean distance (Figure 4b): median distances increased from 20–30 in the test set to 40–60 in CASMI, and the interquartile ranges roughly doubled, from ∼20 to ∼40 units.

**Figure 4:**
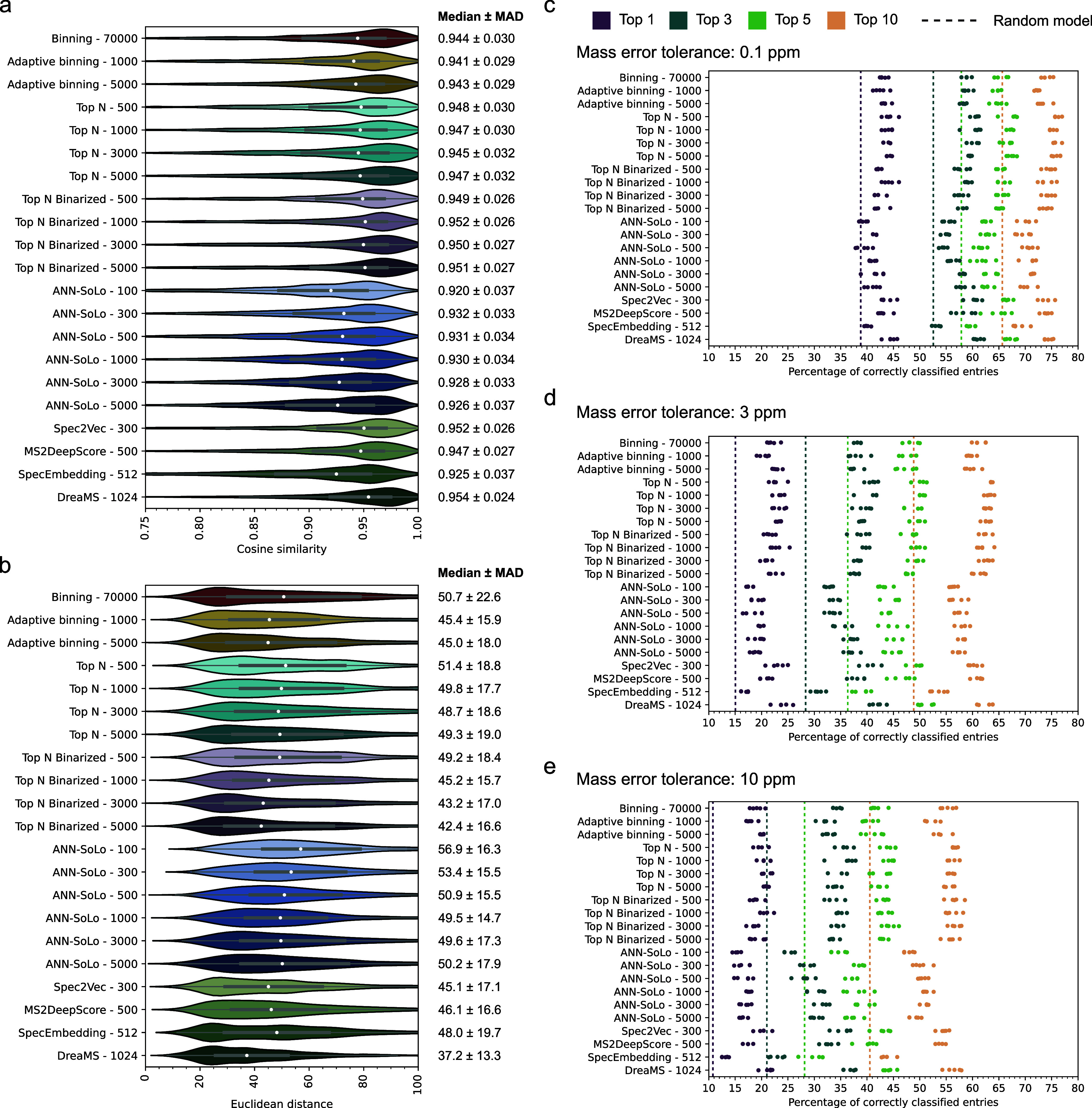
Evaluation of the models’ performance on the CASMI data set (*N* = 304). For each method, all five trained models were used with the same input data. (a,b) Distributions of cosine similarity (a) and Euclidean distance (b) between the predicted and true Mol2Vec embeddings; median and median absolute deviation (MAD) included. (c–e) Retrieval performance when identifying the correct molecular structure from a reference database of ∼0.6 million unique molecules. After filtering candidates by exact mass within (c) 0.1 ppm, (d) 3 ppm, or (e) 10 ppm, we ranked all candidates by cosine similarity to the predicted Mol2Vec embedding and recorded the frequency with which the correct structure (same 14-character InChIKey) appeared in the Top-1, Top-3, Top-5, or Top-10 positions. As a baseline, dashed lines indicate the expected retrieval percentages for a random ranking model, accounting for the varying number of candidates at each ppm tolerance.

In the candidate-retrieval task (Figure 4c–e), several interesting patterns emerged. At 0.1 ppm, Top-1 retrieval rates were similar to those in the test data set; however, the random-model baseline was substantially higher due to the smaller candidate pools at this stringent mass tolerance. As a result, the effective performance gap between the models and the random expectation narrowed considerably: the best models outperformed random by only ∼6%, and the weakest models were indistinguishable from random baseline. This underscores the importance of including a probabilistic baseline to contextualize retrieval percentages.

When widening the mass error tolerance to 3 and 10 ppm, Top-1 retrieval accuracy dropped further, reaching only 10–26%, far below the values observed in the test data set. Yet the relative ranking between methods shifted in interesting ways. Spec2Vec and DreaMS, which previously performed on par with the ANN-SoLo series, retained substantially more accuracy when faced with completely unseen spectra. In fact, within the CASMI2022 data set their performance matched—or exceeded—that of the frequent peak-selection (Top-N and Top-N-binarized) representation. This increased robustness may reflect the extensive pretraining underlying both Spec2Vec and DreaMS, enabling them to generate richer and more informative spectral embeddings even when the input MS/MS data are limited in quality or fragment information.

### Training and Benchmarking Models Using the Spectraverse and MassSpecGym Public Data Sets

To assess the robustness and generalizability of our findings, we applied the same analysis pipeline (spectral featurisation, ANN model training, embedding prediction, and structure retrieval) to two additional, fully open MS/MS data sets: Spectraverse^29^ and MassSpecGym.^28^ For MassSpecGym, we evaluated two data partitioning strategies: the random 80/20 train/validation split used throughout the present study, and the original predefined folds provided with the data set, enabling a direct assessment of how split design influences model performance.

Training and validation loss curves converged smoothly across all featurisation methods for both Spectraverse (Figure S3) and MassSpecGym with the random split (Figure S4), closely mirroring the convergence behavior observed with the primary data set. In both cases, DreaMS and SpecEmbedding achieved the lowest average test MSE, followed by learned embeddings (MS2DeepScore, Spec2Vec) and binning-based methods, with frequent-peaks representations (Top N and Top N Binarized) at low dimensionalities yielding the highest test losses. When training on the MassSpecGym predefined folds (Figure S5), the overall ranking of methods was preserved; however, validation loss curves were substantially noisier and absolute MSE values were markedly higher across all methods. This is expected, as the predefined folds are designed to enforce greater chemical dissimilarity between training and test compounds, constituting a more stringent out-of-distribution evaluation than a random split.

Distributions of cosine similarity and Euclidean distance between predicted and reference Mol2Vec embeddings (Figures S6–S8) reinforced these training observations. For both Spectraverse (Figure S6) and MassSpecGym with the random split (Figure S7), DreaMS and SpecEmbedding produced the tightest cosine similarity distributions, concentrated near 1.0, and the smallest Euclidean distances, reflecting high embedding fidelity. Binning and adaptive binning methods also performed well, with distributions shifted only slightly relative to the learned embeddings. Frequent-peaks methods and low-dimensionality ANN-SoLo showed broader distributions and higher Euclidean distances. Under the predefined MassSpecGym folds (Figure S8), cosine similarity distributions were shifted noticeably toward lower values and Euclidean distances were substantially wider for all methods, quantitatively confirming the greater difficulty of the out-of-distribution split.

Structure retrieval performance on CASMI2022 using models trained on Spectraverse and MassSpecGym (Figures S9–S11) was broadly consistent with the primary data set results. Notably, models trained on the predefined MassSpecGym folds performed closest to the random baseline across most conditions. This is expected, as these folds enforce structural dissimilarity between training and test compounds, placing the models in an out-of-distribution setting where learned relationships transfer poorly to novel chemical space. As a result, performance under predefined splits likely provides a more conservative and realistic estimate of generalization, whereas results obtained with random splits may overestimate real-world annotation performance.

## Discussion

In this work, we systematically evaluated a broad spectrum of MS/MS featurization strategies for machine learning–based metabolite annotation, focusing exclusively on positive-ion protonated adducts. This restriction avoided introducing unnecessary variability in fragmentation behavior across ion types^31^ and ensured sufficient training data density for all feature extraction methods and spectral representations.

An important consideration when interpreting the benchmark results presented here is that the spectral and molecular representations evaluated were not all originally developed with spectra-to-structure retrieval as their primary objective (e.g., Mol2Vec, Spec2Vec, DreaMS). Consequently, underperformance of a given representation on the retrieval task should not be interpreted as a general limitation of that method. That being said, all representations assessed in this study share a common and transferable property: they are featurisation methods that produce fixed-length numerical vectors from mass spectral or molecular data, and as such they are inherently agnostic to the downstream task. The benchmark results reported here therefore characterize the suitability of each representation specifically for embedding-based structure retrieval, and should be contextualised accordingly rather than treated as a comprehensive assessment of the utility of each method across all possible applications.

Conventional fixed binning at 0.01 Da produced competitive performance but remained computationally demanding. With 70,000 input dimensions, most bins are empty, and many correspond to rare fragments that appear infrequently in the spectra. This sparsity makes it difficult for the model to learn meaningful patterns from these bins, limiting its ability to leverage rare but informative fragments. While fixed binning is commonly used, our results suggest that more compact representations can achieve similar or better performance with far greater efficiency.

Adaptive binning mitigated sparsity by constructing equiprobable bins based on the global *m*/*z* distribution. These representations yielded low training errors and strong performance on the test data set, demonstrating that dynamically tuned bin widths help encode spectral information more effectively. However, their performance did not generalize as well to the CASMI2022 data set, where Top-N retrieval accuracy fell behind the frequent peak-selection methods.

Frequent peak-selection methods (Top N and Top N binarized) took the opposite strategy: rather than adjusting bin widths, they reduced sparsity by retaining only the most frequent *m*/*z* values. Despite discarding fewer common peaks, these models delivered robust retrieval performance in the test data set. Interestingly, binarizing intensities improved generalization for test spectra, but not for CASMI2022, where both versions performed similarly.

The ANN-SoLo hashing approach, designed to compress spectra while minimizing dimension, underperformed relative to similarly sized representations. Even with large hashed vectors (3000–5000 elements), mass fragment collisions likely obscured informative spectral features and hindered learning of embedding–structure relationships.

Embedding-based methods showed diverse behavior across data sets and evaluation metrics. Spec2Vec, despite its relatively compact vector size (300 dimensions), achieved competitive performance consistent with the utility of learned peak co-occurrence patterns for spectral representation. MS2DeepScore (500 dimensions), trained on pairwise spectral similarity, performed comparably. SpecEmbedding showed greater variability in downstream CASMI2022 retrieval performance, suggesting a partial decoupling between embedding prediction accuracy and structure retrieval utility. DreaMS produced the most accurate embedding predictions for CASMI2022 in terms of cosine similarity and Euclidean distance, indicating better generalization to spectra produced at a single collision energy. However, improved embedding prediction accuracy did not consistently translate to superior structure retrieval performance. This discrepancy suggests that numerical proximity to the ground-truth Mol2Vec embedding does not guarantee correct candidate ranking, because the Mol2Vec space was not designed to preserve the fine-grained structural distinctions relevant to annotation tasks. Specifically, structurally similar compounds, including stereoisomers, regioisomers, and molecules sharing dominant substructural motifs, may occupy overlapping or indistinguishable regions of the embedding space, making reliable separation during retrieval fundamentally difficult regardless of prediction accuracy.

Furthermore, the pretrained embeddings evaluated in this study (Spec2Vec, MS2DeepScore and DreaMS) differ substantially not only in their architectural design and training objectives, but also in the size and chemical diversity of their pretraining space. DreaMS, by contrast to Spec2Vec and MS2DeepScore, was pretrained on a markedly larger body of mass spectra encompassing a wider range of compound classes and acquisition conditions. Consequently, the degree of chemical overlap between the pretraining corpus of each embedding and the data sets used in the present benchmark is likely to vary considerably across methods, with DreaMS plausibly benefiting from greater representational overlap with compounds included in our evaluation. We did not, however, retrain any learned embedding on our specific benchmark data set. Instead, all learned embeddings are evaluated in their publicly available pretrained forms, which reflects the most realistic deployment scenario for end users. We acknowledge, however, that as new pretrained models are released, the relative performance figures reported here may require updating. To address this as far as possible within the current study, we have structured the accompanying GitHub repository as a modular and extensible pipeline in which any pretrained embedding model can be substituted and evaluated with minimal modification.

Consequently, this asymmetry in pretraining data should be considered when interpreting relative performance differences between learned and nonlearned representations: observed advantages of DreaMS may partly reflect this broader chemical coverage rather than architectural superiority alone. Future benchmarking efforts would benefit from controlled evaluations in which all learned embeddings are retrained on identical data composition.

Furthermore, the better performance on the held-out test data set compared with CASMI2022 data set can be attributed to two contributing factors. First, models trained on merged collision energy spectra may encounter a distribution shift when applied to the single-energy spectra characteristic of CASMI2022, where the input signal is systematically sparser and captures only a subset of the fragment ions present in a merged representation. That said, the structural information encoded in merged spectra is not absent from single-energy spectra but rather distributed across acquisitions, and the most diagnostically informative fragment ions are frequently present at any individual collision energy. Moreover, as community spectral libraries increasingly incorporate multienergy acquisition data, the practical relevance of this limitation is likely to diminish over time. Second, and likely more important, the candidate structures in CASMI2022 are on average less structurally similar to the training set compounds than those in the held-out test data set. Since embedding-based retrieval methods rely on learned structure–spectrum relationships, performance is expected to degrade for compounds whose structural neighborhood is poorly represented in the training data.

The overall retrieval performance across spectral representation types underscores persistent challenges in predicting molecular embeddings with sufficient precision to distinguish closely related structures. We attribute this to three main factors: (i) spectral ambiguity: MS/MS spectra often lack the features required to unambiguously distinguish certain isomers; (ii) training data limitations: even at 71 k molecules, the chemical space covered remains small relative to the full metabolome; (iii) molecular embedding space limitations: Mol2Vec, despite its strengths, can place similar molecules very close together, making small prediction errors lead to incorrect retrievals.

Given these constraints, shifting from full-structure retrieval to substructure-level annotation or class annotation may offer a more attainable goal.^32^ Predicting molecular embeddings could help identify the largest common substructures or functional groups among the top-ranked candidates, which would yield chemically meaningful annotations even when the exact structure cannot be resolved.

This study used a deliberately simple feed-forward ANN architecture to ensure comparability across representation methods, avoid overparameterization, and maintain computational tractability. Future work should explore more sophisticated architectures—such as convolutional, recurrent, or graph-informed neural networks—that may better capture relationships between spectral features and chemical structure.

Similarly, while we focused on Mol2Vec due to its strong performance in previous studies, the benchmarking framework developed here is easily adaptable to other molecular embedding systems. Transformer-based models such as ChemBERTa,^33^ MoLFormer^34^ or MolBERT,^35^ or graph neural network representations, may provide more discriminative embedding spaces for structure retrieval.

Finally, another way to improve specificity in molecule retrieval is to restrict the search space to candidates that are contextually relevant to the sample under study. For example, annotating unknowns in human plasma should rely on human-focused resources such as HMDB^36^ and LIPID MAPS,^37^ whereas plant extracts are better served by natural product–oriented databases like COCONUT^38^ and LOTUS.^39^ Applying a single, broad database such as PubChem to both scenarios increases the number of implausible matches, inflating false discoveries and ultimately reducing retrieval specificity.

## Conclusions

Several practical conclusions emerge for researchers designing or selecting spectral representation pipelines for machine learning–driven metabolomics workflows. First, the choice of featurization strategy has a material and consistent impact on downstream retrieval performance. Among the methods evaluated, DreaMS, adaptive binning, and frequent peak-selection approaches delivered the strongest and most consistent results across data sets and mass tolerance conditions. Second, vector dimensionality matters, but only up to a point. For methods where dimensionality is a configurable parameter, performance improved with increasing vector size up to a saturation threshold, beyond which additional dimensions provided no clear benefit. Third, retrieval accuracy is strongly dependent on mass precision. Performance declined substantially as mass tolerance was widened from 0.1 to 10 ppm, reflecting the well-established relationship between candidate set size and ranking difficulty, highlighting that reported benchmark figures should always be interpreted alongside the ppm tolerance at which they were obtained. Fourth, embedding prediction accuracy and structure retrieval performance are not interchangeable metrics. Methods achieving low mean squared error in Mol2Vec prediction did not always produce superior candidate rankings, because the Mol2Vec embedding space does not preserve the fine-grained structural distinctions, such as stereoisomers or regioisomers, that are most critical for correct annotation. Fifth, data partitioning strategy has a substantial effect on reported performance. Results obtained under random train/test splits likely overestimate real-world generalization to novel chemical space. Evaluations conducted under structurally dissimilar splits, as exemplified by the MassSpecGym predefined folds, provide more conservative estimates of annotation performance. We encourage future benchmarking studies to report results under both random and dissimilarity-based splits. Finally, all code, trained models, and data required to reproduce and extend this benchmark are publicly available, and the modular pipeline is designed to accommodate newly released pretrained models and alternative molecular embedding targets as the field continues to evolve.

## Supplementary Material





## Data Availability

Code and data availability: All scripts, and reproduction instructions are available at GitHub repository https://github.com/RogerGinBer/benchmark_ms2_featurization. Public spectra used in the analysis (MassSpecGym, Spectraverse, and CASMI2022), precalculated features, trained ANN models and raw experimental figures are archived on Zenodo (10.5281/zenodo.17866068), alongside descriptive metadata and guidance on deposited files. Computational workflows are documented as Jupyter notebooks covering data preprocessing, model training, and embedding evaluation. Supporting Table 1 provides InChIKey identifiers, source database names, and fold membership annotations for every spectrum in the training and test splits, enabling reconstruction of the exact partitioning used in this study by readers with access to the relevant proprietary libraries.

## Notes

Analysis of the spectral database metadata (precursor *m*/*z* and ClassyFire-predicted chemical ontology classes) in the train/test splits used in the experiment (Figure S1); number of candidate structures found at different mass tolerance thresholds in the retrieval task (Figure S2); ANN training results (Figures S3–S5), cosine and euclidean distances between predictions and actual vectors (Figures S6–S8) and database retrieval (Top N) results (Figures S9–S11) for the Spectraverse and MassSpecGym (original and random folds) data sets (PDF)
Metadata and identifiers of the molecular structures present in the spectral database, including original database source, and to which split they belonged in each of the cross-validation folds (CSV)
