# Supplementary material for: Benchmarking MS/MS Featurization Strategies for Machine Learning-Driven Metabolite Structure Annotation
Source: J Am Soc Mass Spectrom. 2026 Jun 15;37(7):1550–61. doi: 10.1021/jasms.5c00428 (PMC13329996; doi:10.1021/jasms.5c00428)
Supplement: Supplementary file 1 [file js5c00428_si_001.pdf]

Supporting Information

Benchmarking MS/MS featurization strategies for machine learning-driven metabolite structure annotation

Roger Giné[1,2], Ivan Pérez-López[1], Josep Mª Badia[1], Jordi Capellades[2,3], Oscar Yanes[1,2,3]*

1. Universitat Rovira i Virgili, Department of Electronic Engineering, 43007 Tarragona, Spain.
2. CIBER de Diabetes y Enfermedades Metabólicas Asociadas (CIBERDEM), Instituto de Salud Carlos III, 28029 Madrid, Spain.
3. Metabolomics Platform, Institut de Recerca Biomèdica Catalunya Sud, Hospital Universitari Sant Joan de Reus, 43204 Reus, Spain

*Corresponding author. Email address: oscar.yanes@urv.cat

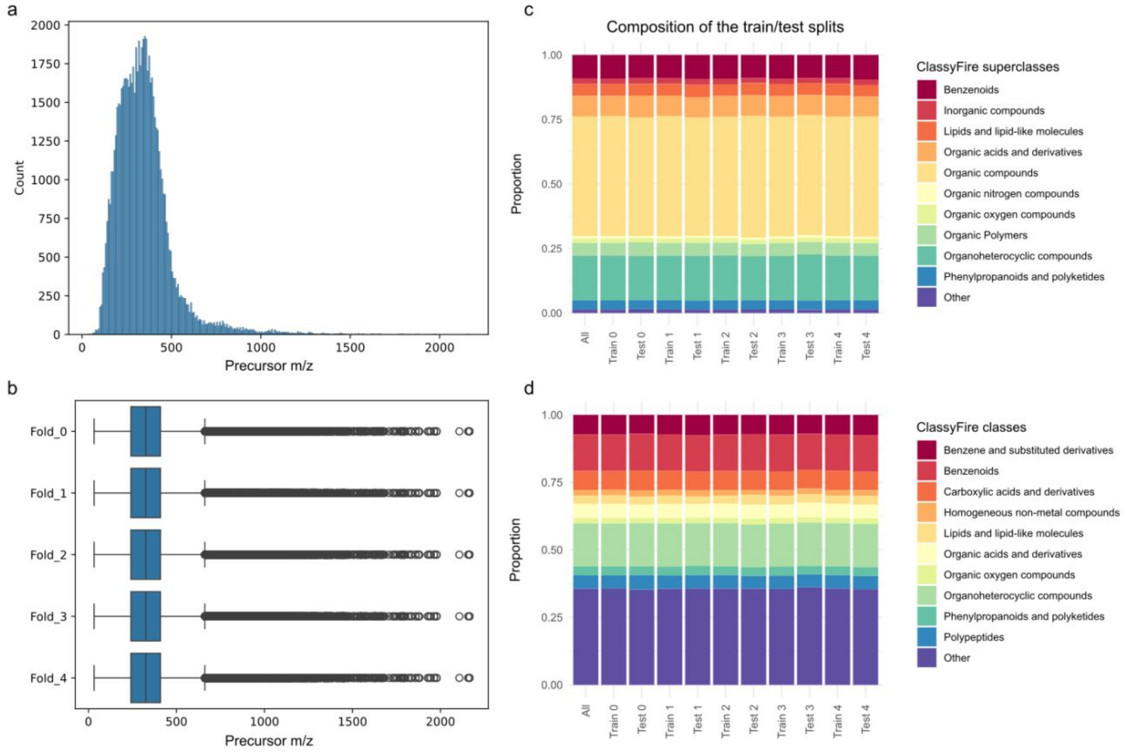

Figure S1. Metadata analysis of the compounds from our in-house spectral database. (a) Global distribution if precursor m/z values in the spectral database. (b) Distribution of precursor m/z values across the training splits for each of the five folds in the spectral dataset. (c) and (d) Proportions of the major ClassyFire superclasses and classes of the molecular structures present in the full spectral database and across the five train and test splits. No significant compositional differences were observed between the training and testing folds using a Chi-squared statistical test.

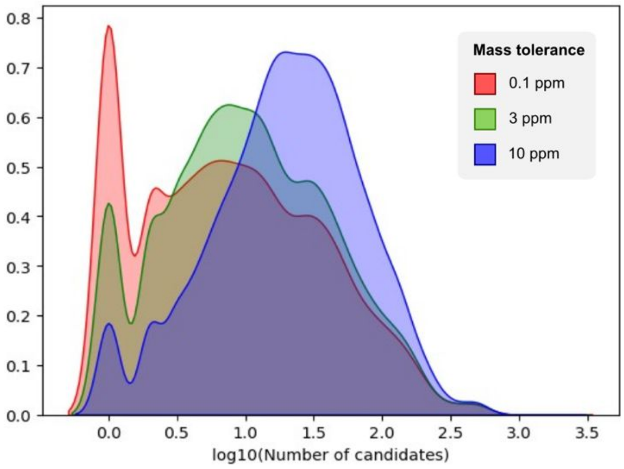

Figure S2. Distribution of the number of candidate structures retrieved from the 0.6M molecular structure database for test fold entries, as a function of the maximum precursor mass tolerance (in parts per million, ppm). Median candidate counts are 7, 10, and 21 for mass tolerances of 0.1, 3, and 10 ppm, respectively.

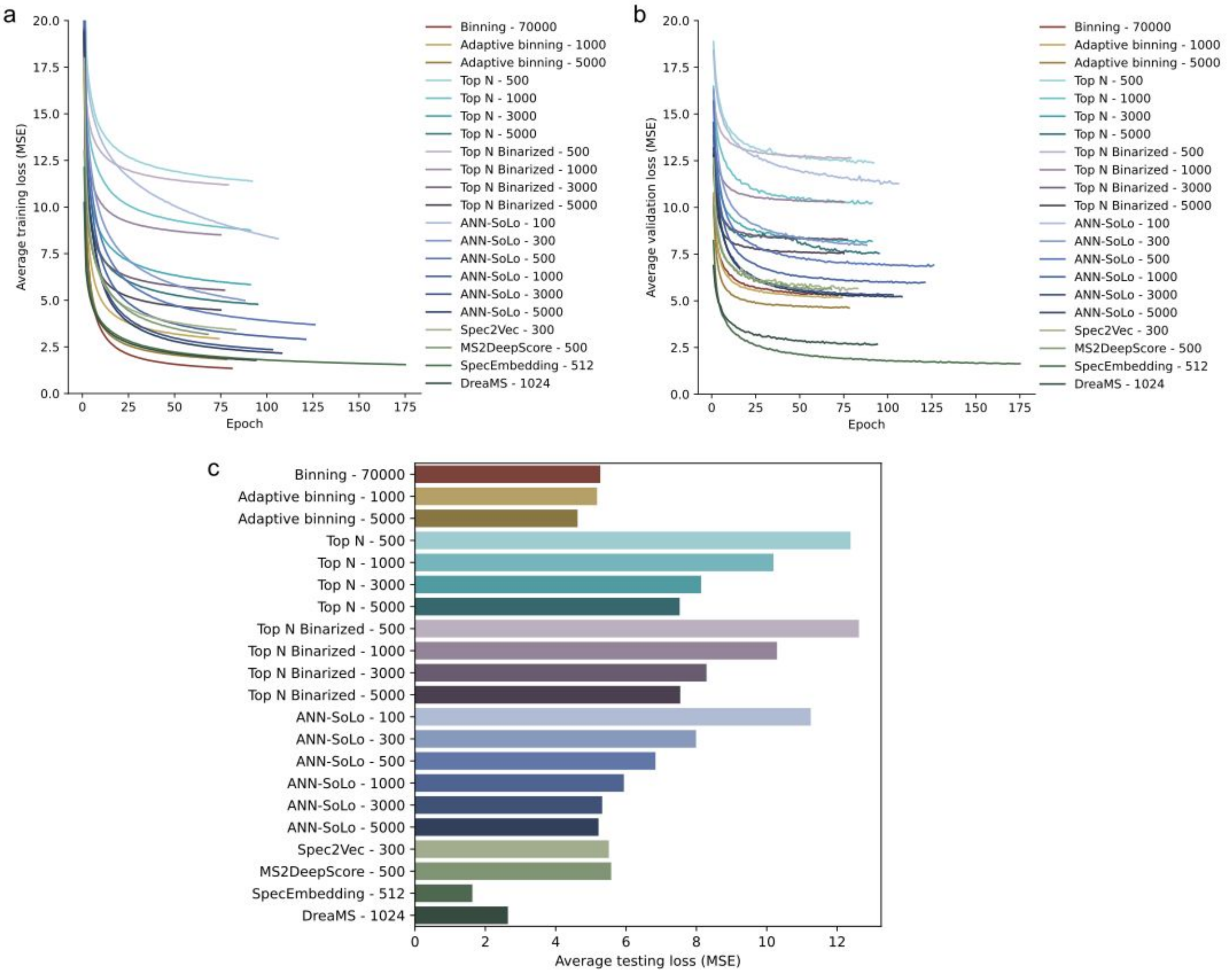

Figure S3. Training results obtained using the Spectraverse dataset. (a) Training loss (mean squared error, MSE) across epochs. (b) Validation loss (MSE) across epochs. (c) Average test loss (MSE) computed on the held-out fold using the trained models.

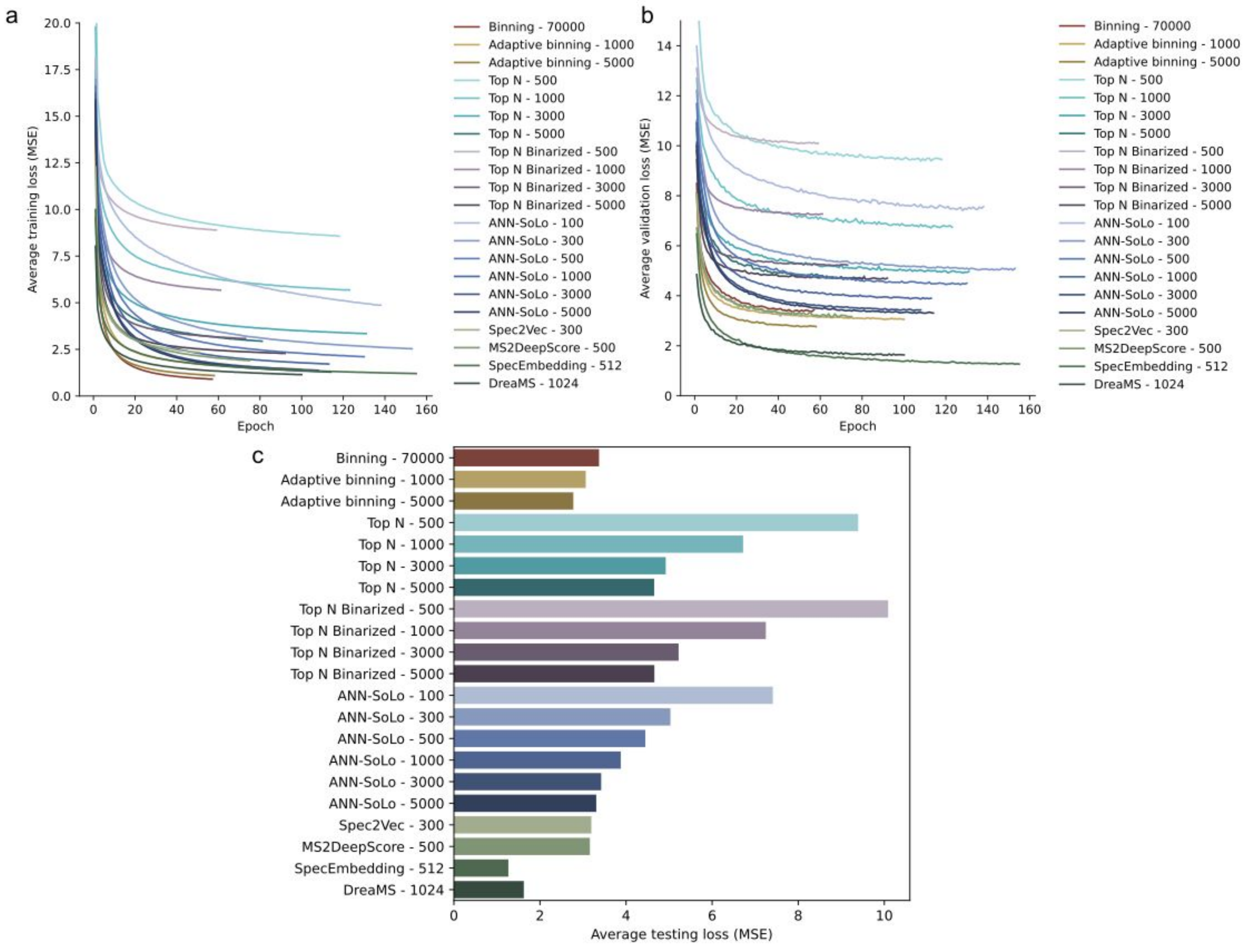

Figure S4. Training results obtained using the MassSpecGym dataset with a random 80/20 train/validation split. (a) Training loss (mean squared error, MSE) across epochs. (b) Validation loss (MSE) across epochs. (c) Average test loss (MSE) computed on the held-out fold using the trained models.

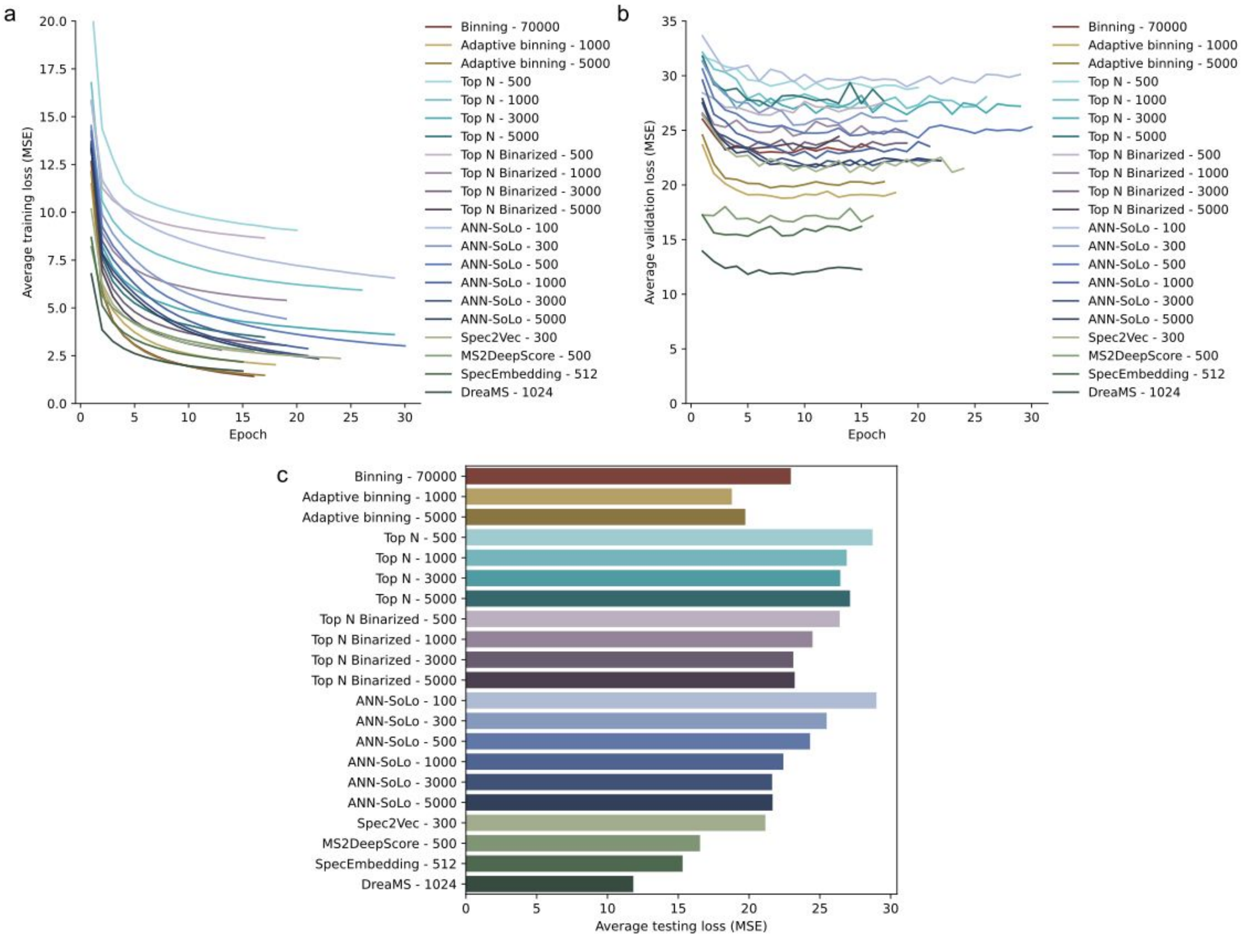

Figure S5. Training results obtained using the MassSpecGym dataset with the original predefined folds as described in the dataset. (a) Training loss (MSE) across training epochs. (b) Validation loss (MSE) across training epochs. (c) Mean MSE on the held-out fold using the trained models.

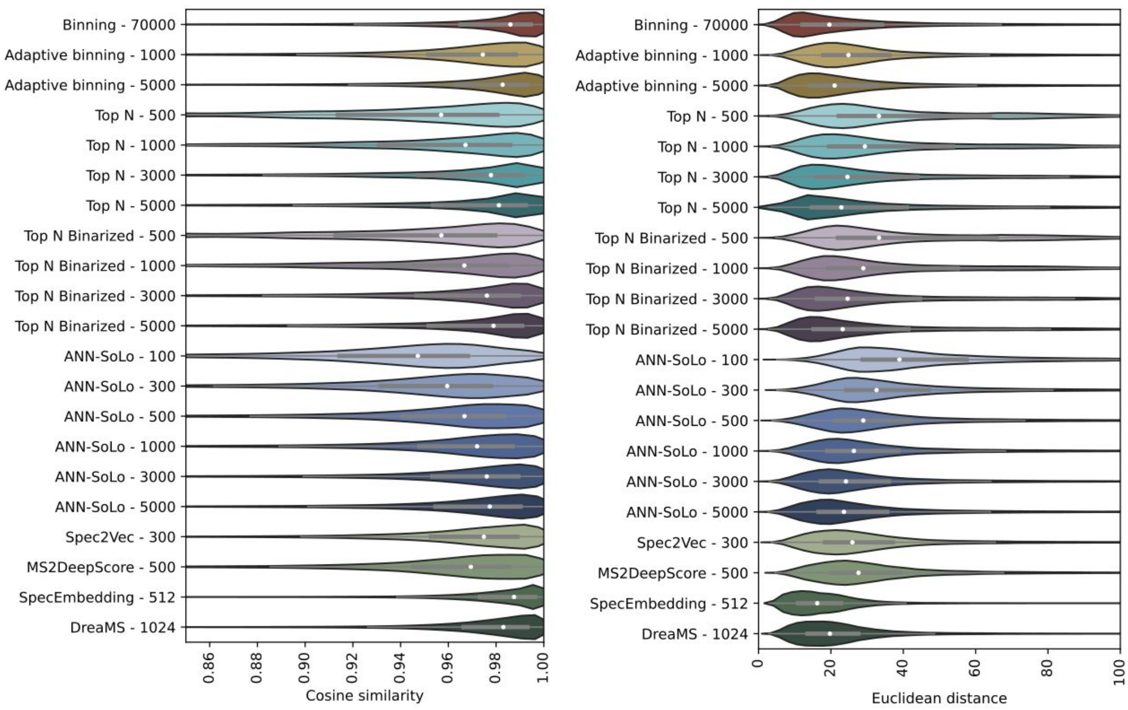

Figure S6. Distributions of cosine similarity and Euclidean distance between predicted and reference Mol2Vec embeddings for entries in the Spectraverse test split.

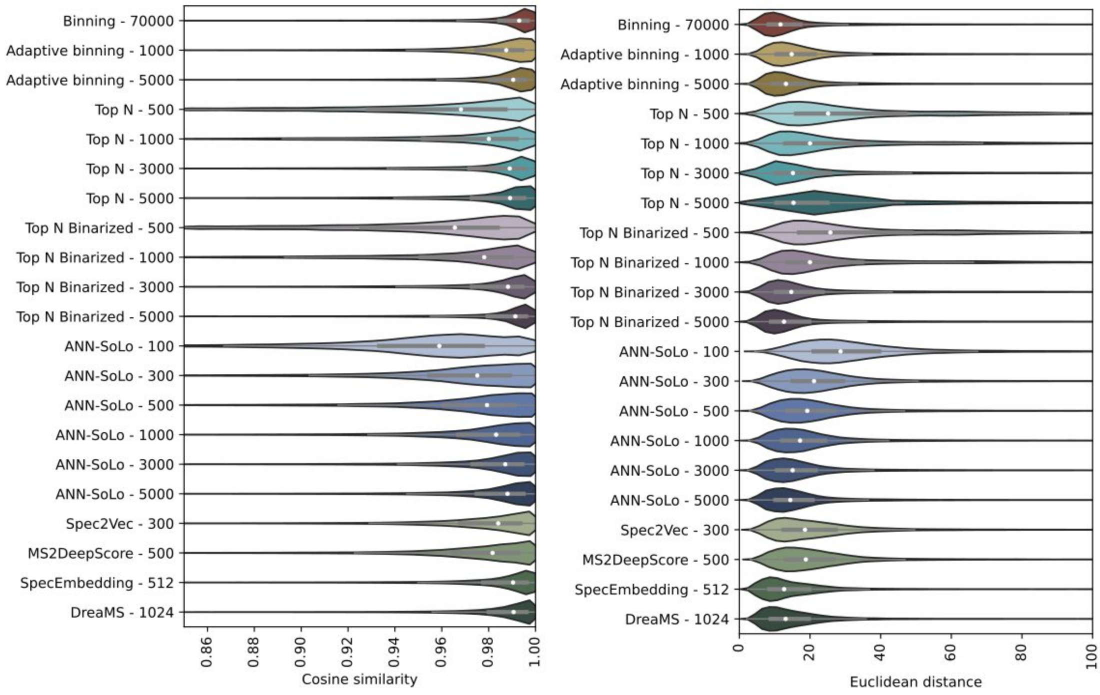

Figure S7. Distributions of cosine similarity and Euclidean distance between predicted and reference Mol2Vec embeddings for entries in the MassSpecGym with a random 80/20 train/test split.

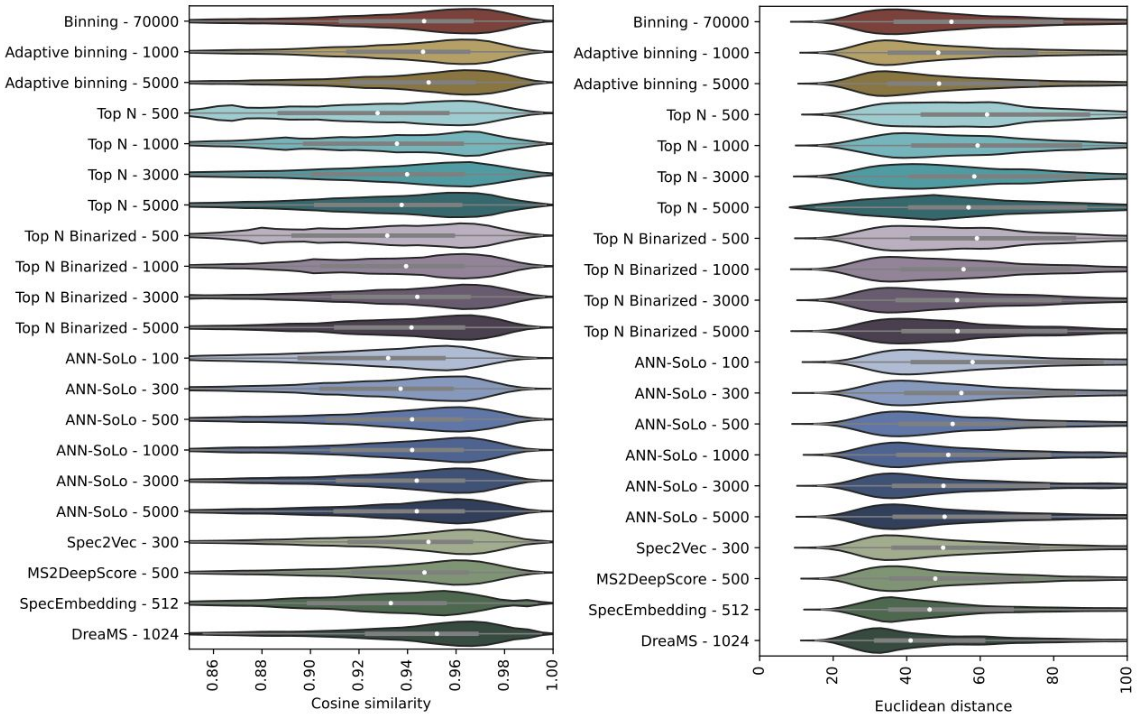

Figure S8. Distributions of cosine similarity and Euclidean distance between predicted and reference Mol2Vec embeddings for entries in the MassSpecGym test split with the original predefined folds as described in the dataset.

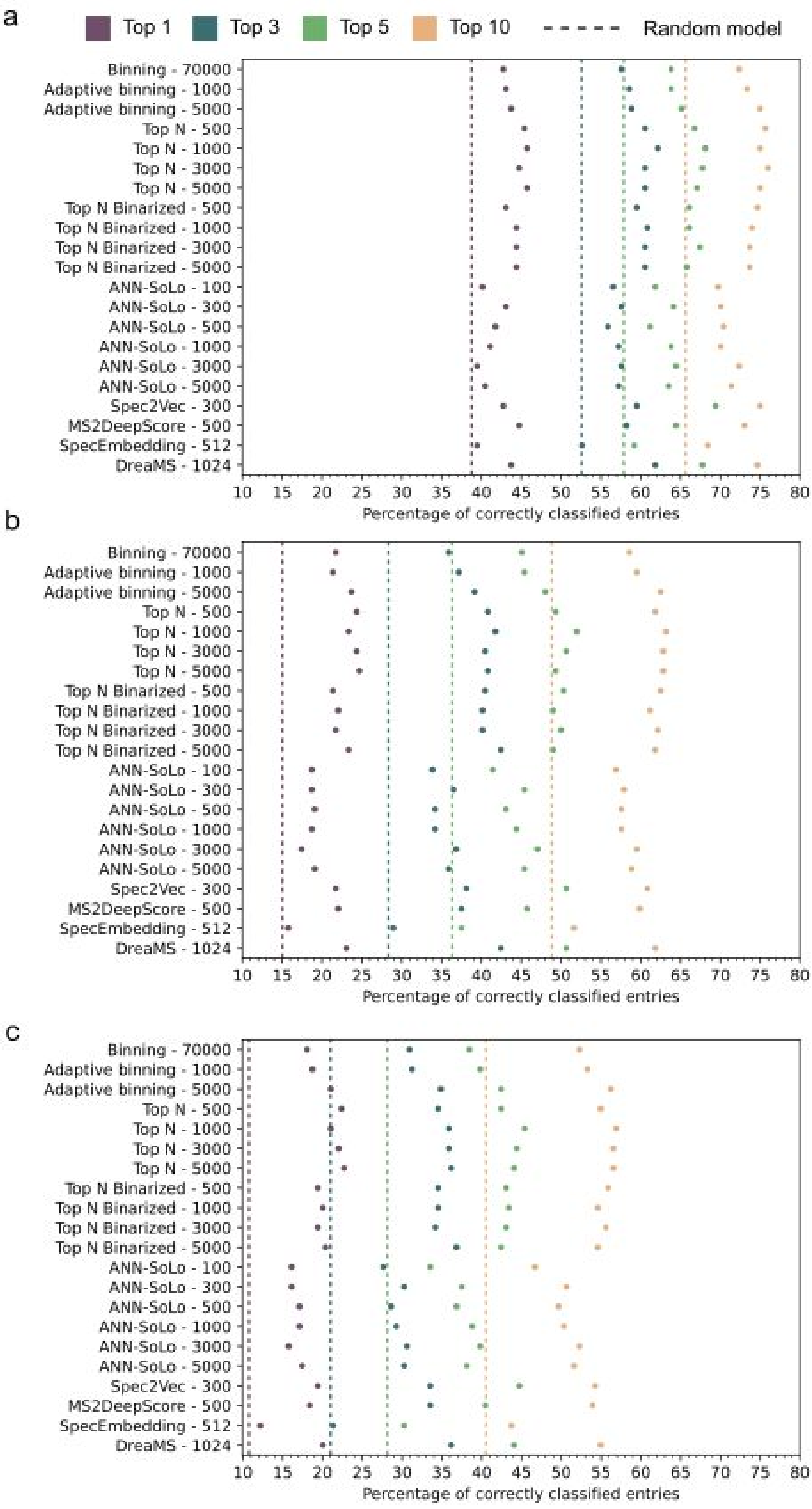

Figure S9. Molecule retrieval performance of Spectraverse-trained models on CASMI22 at Top-1, Top-3, Top-5, and Top-10. (a) 0.1 ppm mass tolerance; (b) 3 ppm mass tolerance; (c) 10 ppm mass tolerance. As a baseline, dashed lines indicate the expected retrieval percentages for a random ranking model, accounting for the varying number of candidates at each ppm tolerance.

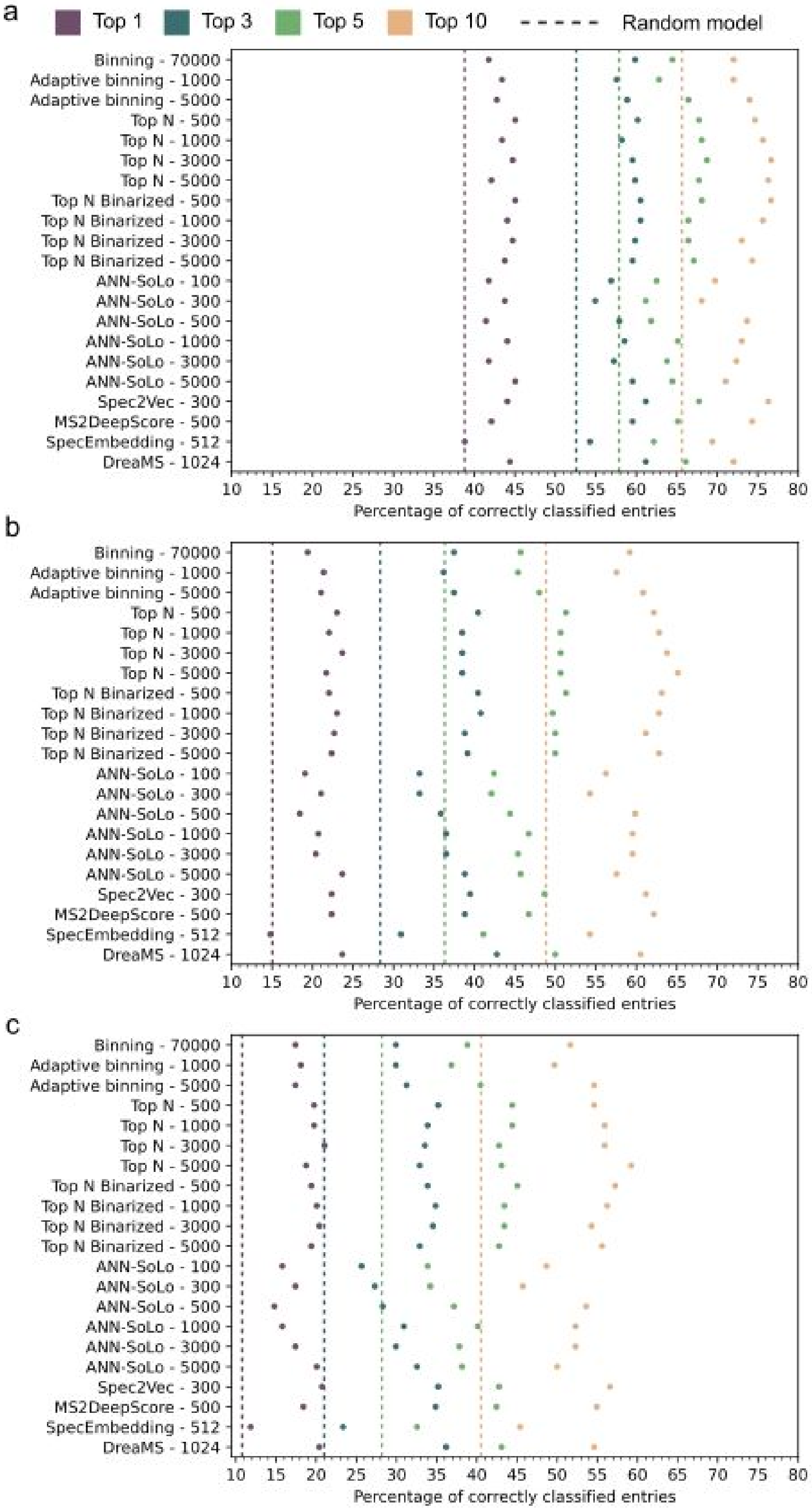

Figure S10. Molecule retrieval performance of MassSpecGym-trained models (with a random 80/20 train/test split) on CASMI22 at Top-1, Top-3, Top-5, and Top-10. (a) 0.1 ppm mass tolerance; (b) 3 ppm mass tolerance; (c) 10 ppm mass tolerance. As a baseline, dashed lines indicate the expected retrieval percentages for a random ranking model, accounting for the varying number of candidates at each ppm tolerance.

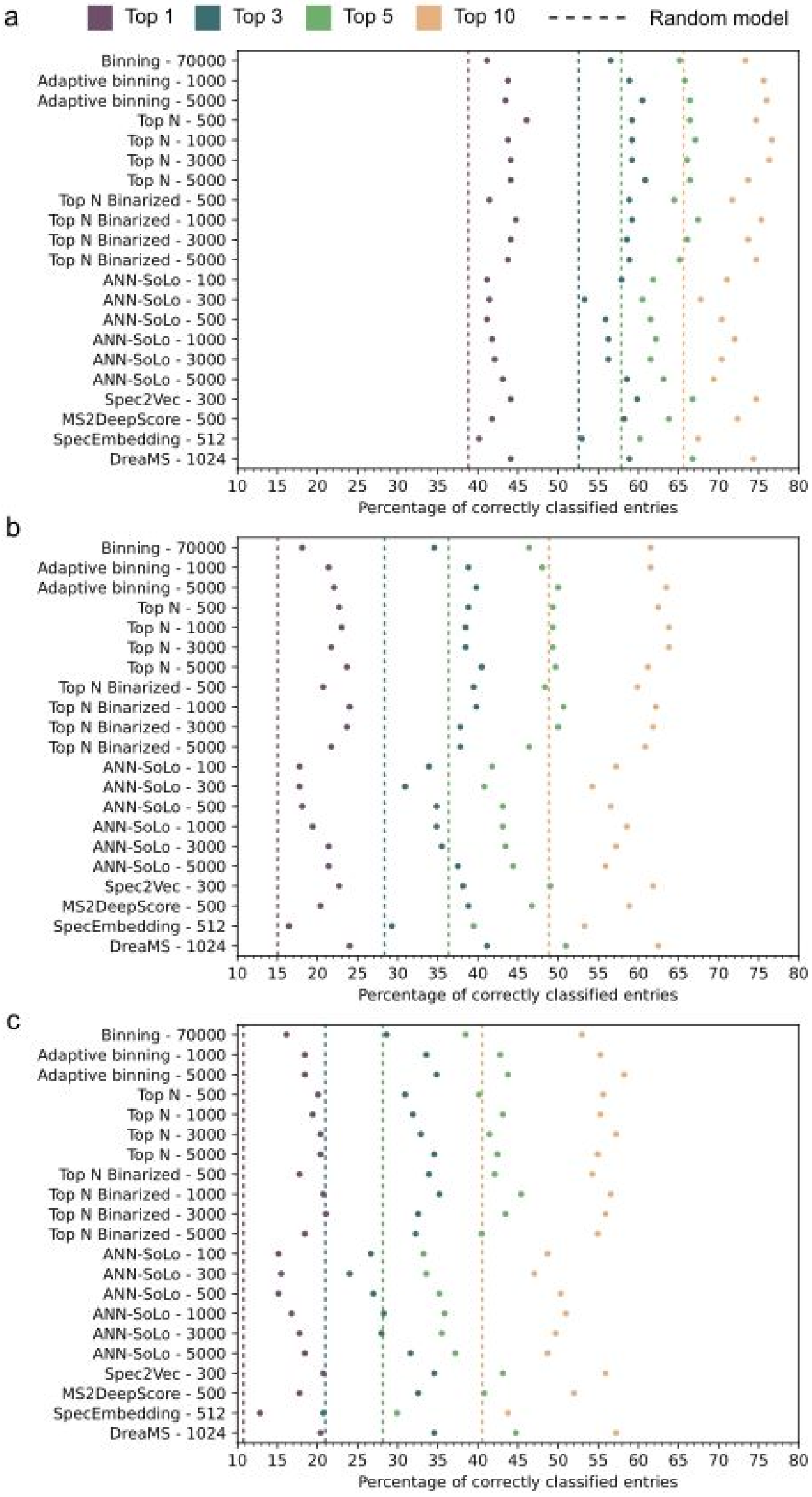

Figure S11. Molecule retrieval performance of MassSpecGym-trained models (test split with the original predefined folds as described in the dataset) on CASMI22 at Top-1, Top-3, Top-5, and Top-10. (a) 0.1 ppm mass tolerance; (b) 3 ppm mass tolerance; (c) 10 ppm mass tolerance. As a baseline, dashed lines indicate the expected retrieval percentages for a random ranking model, accounting for the varying number of candidates at each ppm tolerance.
